# Transcription Factor Nrf2: a novel target to modulate inflammatory and neuroprotective responses in Parkinson’s disease

**DOI:** 10.1186/2193-1801-4-S1-L43

**Published:** 2015-06-12

**Authors:** Antonio Cuadrado

**Affiliations:** Centro de Investigación en Red sobre Enfermedades Neurodegenerativas (CIBERNED), Instituto de Investigación Sanitaria La Paz (IdiPAZ). Departamento de Bioquímica e Instituto de Investigaciones Biomédicas “Alberto Sols” CSIC-UAM, Facultad de Medicina, Universidad Autónoma de Madrid, Spain

**Keywords:** neuroinflammation, Nrf2, Parkinson’s disease

Stopping the succession of events that lead to development of Parkinson’s disease (PD) is a principal challenge that requires a brain protective approach in early diagnosed patients. Although PD ethiopathology may not have a single causative factor, information from sporadic and familial cases together with chemical and genetic animal models strongly suggests that low-grade chronic inflammation and oxidative stress play a critical role. Our team is studying the relevance of the transcription factor NRF2 (Nuclear factor (erythroid-derived 2)-like 2), a master regulator of oxidant and inflammatory defense, as a new therapeutic target in PD. The pro-inflammatory activation of microglia and astroglia in response to LPS, MPTP and human α-synuclein over-expression is exacerbated in Nrf2-deficient mice, thus demonstrating an immunomodulatory role of this protein. In PD patients, some evidence gathered from epidemiological, genetic and anatomopathologic studies also support a protective role of NRF2. Several compounds activate NRF2 and provide an immunomodulator and cytoprotective response in preclinical animal models of PD. At this time a crucial point to translate these promising results to the clinic is the discovery of a drug with good pharmacokinetics and pharmacodynamics that fulfill criteria of safety, tolerability and efficacy. The use of repurposing drugs, such as dimethyl fumarate used nowadays for treatment of remitting relapsing multiple sclerosis, may provide excellent candidates.

